# Commentary: Altered sleep composition after traumatic brain injury does not affect declarative sleep-dependent memory consolidation

**DOI:** 10.3389/fnhum.2015.00379

**Published:** 2015-06-30

**Authors:** Simon J. Durrant

**Affiliations:** Sleep and Cognition Laboratory, School of Psychology, University of LincolnLincoln, UK

**Keywords:** sleep, memory deficit, memory consolidation, TBI, trauamtic brain injury

## Sleep disturbance without memory deficit in TBI: a compensatory mechanism?

Traumatic head injury (TBI) brings with it a diverse array of symptoms and co-morbid conditions, with memory deficits and sleep disturbances amongst the most highly reported (Bhalerao et al., 2013). Recent studies of TBI inpatients found that a clear majority of TBI patients had disturbed sleep patterns (Makley et al., 2007) and that a return to normal sleep was associated with a reduction of memory impairment (Makley et al., 2009). However, 6 months or more after TBI as many as half of the patients still report significant sleep disturbance (Baumann et al., 2007). This raises the possibility that the longer-term memory impairment commonly seen in TBI (Ruttan et al., 2008) may be associated with continuing sleep disturbance.

In recent years, a wealth of evidence has accumulated linking sleep to memory consolidation (Rasch and Born, 2013). In spite of this, until now there has been almost no investigation of the role of sleep disturbance in memory deficit where the sleep disturbance occurs as a result of another condition. Where such studies have occurred, they have typically compared disturbed and non-disturbed sleep groups suffering from the underlying condition (e.g., Wilde et al., 2007, in the case of TBI) rather than evaluating the effect of sleep on memory directly in both patients and matched healthy controls using a sleep/wake design. As such, the new study from Mantua et al. (2015) is especially welcome and will hopefully herald a new era of investigation into the contribution of co-morbid sleep disturbances to memory deficits not only in TBI, but a wide variety of other conditions where disturbed sleep and reduced memory are present (such as Alzheimer's Disease and Parkinson's Disease, to give two obvious examples).

The central finding of the study—that sleep-dependent consolidation remains fully intact in the TBI participants in spite of disturbed sleep—is highly informative. It tells us that, even when tests fail to detect a memory deficit, it may be because other mechanisms—in this case an increase in slow wave sleep (SWS)—compensate for the deficit, in a similar way that slowed responses in TBI patients may be used to compensate for other cognitive deficits (Ozen and Fernandes, 2012).

## Integrating medical conditions, sleep disturbances and memory deficits in one model

As the first example of what may become a new field of research, this study has much to recommend it by way of methodology. The use of gold-standard polysomnography rather than actigraphy or subjective questionnaires allows an examination of sleep structure rather than simply duration or sleep pattern, and the results of this study highlight how important this is, not least because objective sleep abnormalities in TBI do not necessarily relate to subjectively-reported sleep quality (Arbour et al., 2015). Similarly, the inclusion of matched healthy controls means that not only can we understand the role of sleep in memory consolidation for the TBI participants, but we can also know whether or not that differs from the wider population.

There are also areas that future studies may wish to develop. The authors acknowledge a number of limitations of this first study, and amongst these the heterogeneity of the sample stands out. Clearly a more fine-grained approach to inclusion could yield additional insights for clinicians as to exactly which patients are likely to be suffering from a sleep-related memory deficit.

Finally, there remains the important and thorny topic of causality. (Mantua et al., 2015) following common practice in sleep and memory research, looked for correlations between sleep parameters and memory performance, having initially ruled out working memory differences between TBI and non-TBI groups. This is a perfectly valid approach and certainly one that makes sense for a first investigation. Fundamentally, though, studies in this area are really interested in a three-way relationship, which could best be characterized on continua in all respects: the severity of the underlying condition (TBI in this case), the nature of the co-morbid sleep disturbance, and the extent of the memory deficit. Investigating pairwise relationships is certainly informative, but it does not tell us the structure of the relationship between the three entities. For example, it can be argued that the present study implies that TBI causes sleep disturbance, and sleep disturbance causes memory deficit. This is certainly a plausible hypothesis, but it may equally be the case that TBI causes both the sleep disturbance and the memory deficit, and the correlation between the latter two is illusory. Fortunately, modern statistics provides us with methods equal to the task of disentangling such relationships, through techniques such as structural equation modeling. Future studies designed around such techniques could go a long way toward highlighting the pattern of causality in this area.

This new study represents a fine contribution in its own right, but hopefully its biggest legacy will be to stimulate a new area of research into the mediating role of sleep in memory deficits associated with a wide variety of medical conditions. A better understanding of the relationship between these is an essential step in developing better treatments. The study by Mantua et al. (2015) is an excellent start.

### Conflict of interest statement

The author declares that the research was conducted in the absence of any commercial or financial relationships that could be construed as a potential conflict of interest.

## References

[B1] Arbour C. Khoury S. Lavigne G. J. Gagnon K. Poirier G. Montplaisir J. Y.. (2015). Are NREM sleep characteristics associated to subjective sleep complaints after mild traumatic brain injury? Sleep Med. 16, 534–539. 10.1016/j.sleep.2014.12.002 25747335

[B2] Baumann C. R. Werth E. Stocker R. Ludwig S. Bassetti C. L. (2007). Sleep-wake disturbances 6 months after traumatic brain injury: a prospective study. Brain 130, 1873–1883. 10.1093/brain/awm109 17584779

[B3] Bhalerao S. U. Geurtjens C. Thomas G. R. Kitamura C. R. Zhou C. Marlborough M. (2013). Understanding the neuropsychiatric consequences associated with significant traumatic brain injury. Brain Inj. 27, 767–774. 10.3109/02699052.2013.793396 23789861

[B4] Makley M. J. English J. B. Drubach D. A. Kreuz A. J. Celnik P. A. Tarwater P. M. (2007). Prevalence of sleep disturbance in closed head injury patients in a rehabilitation unit. Neurorehabil. Neural Repair 22, 341–347. 10.1177/1545968308315598 18663247

[B5] Makley M. J. Johnson-Greene L. Tarwater P. M. Kreuz A. J. Spiro J. Rao V.. (2009). Return of memory and sleep efficiency following moderate to severe closed head injury. Neurorehabil. Neural Repair 23, 320–326. 10.1177/1545968308325268 19171947

[B6] Mantua J. Mahan K. M. Henry O. S. Spencer R. M. C. (2015). Altered sleep composition after traumatic brain injury does not affect declarative sleep-dependent memory consolidation. Front. Hum. Neurosci. 9:328. 10.3389/fnhum.2015.00328 26097451 PMC4456580

[B7] Ozen L. J. Fernandes M. A. (2012). Slowing down after a mild traumatic brain injury: a strategy to improve cognitive task performance? Arch. Clin. Neuropsychol. 27, 85–100. 10.1093/arclin/acr087 22068441

[B8] Rasch B. Born J. (2013). About sleep's role in memory. Physiol. Rev. 93, 681–766. 10.1152/physrev.00032.2012 23589831 PMC3768102

[B9] Ruttan L. Martin K. Liu A. Colella B. Green R. E. (2008). Long-term cognitive outcome in moderate to severe traumatic brain injury: a meta-analysis examining timed and untimed tests at 1 and 4.5 or more years after injury. Arch. Phys. Med. Rehabil. 89, S69–S76. 10.1016/j.apmr.2008.07.007 19081444

[B10] Wilde M. C. Castriotta R. J. Lai J. M. Atanasov S. Masel B. E. Kuna S. T. (2007). Cognitive impairment in patients with traumatic brain injury and obstructive sleep apnea. Arch. Phys. Med. Rehabil. 88, 1284–1288. 10.1016/j.apmr.2007.07.012 17908570

